# Transcatheter Tricuspid Valve Clinical Trials: Incomplete Data and FDA-Approved Devices

**DOI:** 10.1016/j.shj.2024.100335

**Published:** 2024-07-15

**Authors:** Deborah Furman, Brian Whisenant

**Affiliations:** a Department of Internal Medicine, University of Utah, Salt Lake City, UT; b Department of Cardiovascular Medicine, Intermountain Medical Center, Murray, UT

**Keywords:** EVOQUE, Transcatheter, TriClip, Tricuspid regurgitation, TRILUMINATE, TRISCEND

## Abstract

Each reviewed trial of transcatheter tricuspid valve intervention demonstrated clinically meaningful improvement in Kansas City Cardiomyopathy Questionnaire (KCCQ)-defined quality of life and favorable right ventricular remodeling. KCCQ correlates with tricuspid regurgitation (TR) reduction, heart failure hospitalization, and mortality. Change in KCCQ is therefore meaningful both as a measure of quality of life and as a surrogate endpoint of the impact of TV interventions. TRILUMINATE, the first randomized trial to evaluate the safety and efficacy of tricuspid edge-to-edge repair, demonstrated clinically important benefits in KCCQ score and favorable right ventricular remodeling, which are appropriate endpoints for this symptomatic population. TRISCEND II, which evaluated the safety and effectiveness of the EVOQUE valve, enrolled patients with more New York Heart Association class III and IV heart failure and lower KCCQ scores than TRILUMINATE. EVOQUE tricuspid valve replacement in TRISCEND II reduced TR to mild or less in 94% of patients compared to 50% of patients treated with TriClip in TRILUMINATE. The higher-risk TRISCEND II population and the near elimination of TR with EVOQUE are consistent with the favorable trend in EVOQUE mortality. Patients with diminished left ventricular systolic function being considered for either of these tricuspid valve interventions should be optimized with guideline directed medical therapy. Significant left-side valve disease should be treated. Patients should be optimally diuresed and excluded with severe pulmonary hypertension. Patients with persistent severe TR and symptoms or right ventricular enlargement should be considered for intervention. Smaller coaptation gaps without significant pacemaker impingement may be well served with transcatheter tricuspid edge-to-edge repair, while larger coaptation gaps and leaflets pinned by right ventricular leads, particularly in patients tolerating oral anticoagulation, may be best served with transcatheter tricuspid valve replacement.

## Highlights

• Transcatheter TR studies have consistently demonstrated favorable RV remodeling.
• TRILUMINATE was underpowered for heart failure hospitalization and mortality.
• KCCQ gains correlate with TR Reduction, HF hospitalization and mortality.
• Full results of the higher risk TRISCEND II population are eagerly awaited.

## Introduction

**Figure 1:**
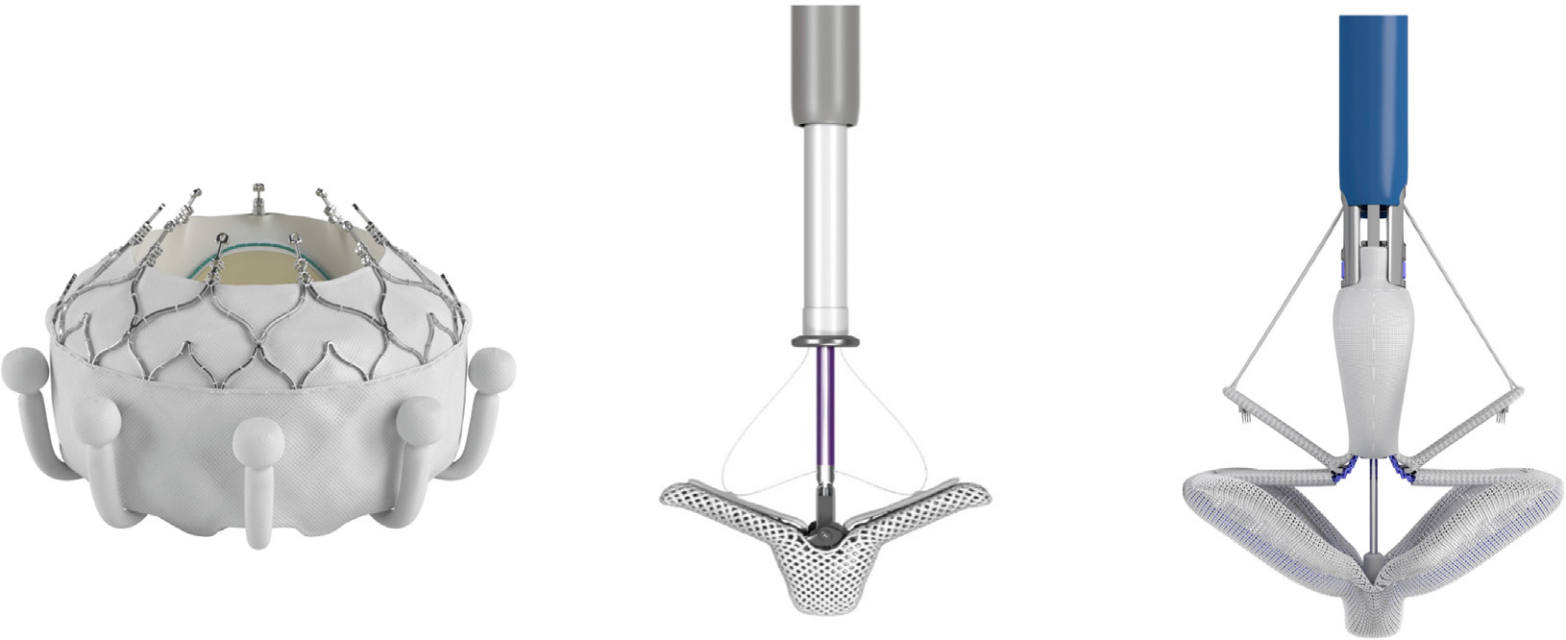
Investigational and commercial devices for transcatheter tricuspid valve repair or replacement. From left to right, the EVOQUE tricuspid valve replacement system, the TriClip transcatheter tricuspid valve repair system, and the PASCAL transcatheter valve repair system.

Driven by a large unmet clinical need, the tricuspid valve has been a major cardiology research focus of the past few years, which is now coming to fruition. On February 2, 2024, the EVOQUE Tricuspid Valve Replacement System (Edwards Lifesciences, Irvine, CA) received approval from the United States FDA. This was soon followed by the TriClip (Abbott Medical, Abbott Park, IL) which received FDA approval on April 2, 2024 (Figure 1).

**Figure 2:**
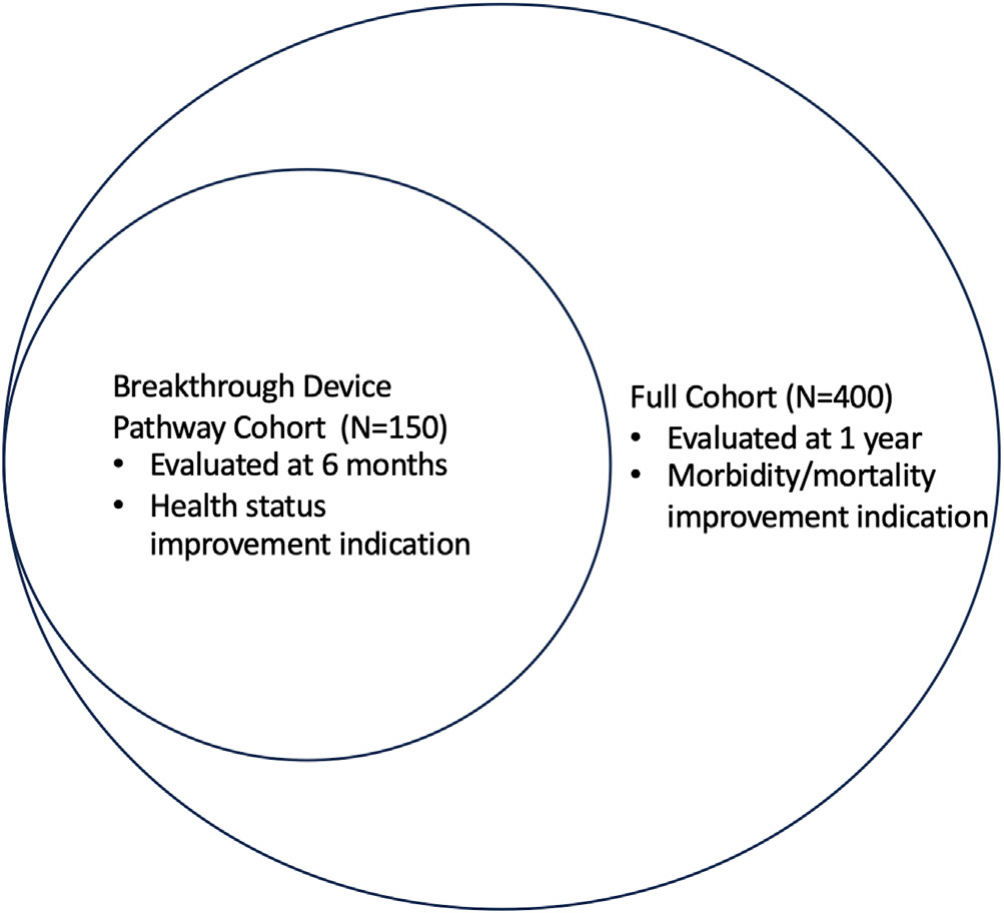
Construct of the TRISCEND II Trial Data Analysis Plan. This figure illustrates the 2-phase design of the TRISCEND II pivotal trial, which consists of 30-day safety and 6-month effectiveness endpoints for the first 150 patients, followed by a hierarchical composite endpoint of 1-year safety and effectiveness for the full 400-patient cohort in the second phase. https://www.accessdata.fda.gov/cdrh_docs/pdf23/P230013B.pdf.

The initial 350 patient results of the TRILUMINATE Trial of TriClip transcatheter tricuspid valve repair were presented at the American College of Cardiology meeting in March 2023, with simultaneous publication in the New England Journal of Medicine.^1^ This was supplemented with a presentation of the entire 572 patient cohort in October 2023 at the Transcatheter Cardiovascular Therapeutics (TCT) conference.^2^ An FDA panel convened on February 13, 2024, to review the TRILUMINATE trial with online publication of additional TRILUMINATE trial outcomes.^3^ The EVOQUE valve received U.S. FDA Breakthrough Device Designation, which is designed to provide patients with potentially earlier access to new therapies by expediting development, assessment, and review (Figure 2). The results of the breakthrough cohort were also presented at TCT in October 2023.^4^ Additional data from the breakthrough and full cohorts is available in the instructions for use, which accompanied approval of the EVOQUE valve.^5^ The full cohort of TRISCEND II pivotal trial patients is expected to be presented in late 2024. The PASCAL Transcatheter Valve Repair System (Edwards Lifesciences, Irvine, CA) (Figure 1) is currently being evaluated in the CLASP II TR Trial (NCT04097145). We review key transcatheter tricuspid regurgitation (TR) data and provide a perspective on treatment indications along with the anticipated risks and benefits of competing technologies.

## Tricuspid Regurgitation

Clinically significant TR is common, increases with age, and is associated with impaired quality of life and excess mortality.^6^ TR can be primary, relating to the structural apparatus of the valve, or secondary, in which valve incompetence is a consequence of annular dilation and/or leaflet tethering. Primary TR arises from congenital and acquired anatomic abnormalities. Acquired causes of primary TR include infective endocarditis, infiltrative causes such as sarcoidosis, and more commonly in developing countries, rheumatic disease. Additionally, iatrogenic TR related to pacemaker leads traversing the tricuspid valve or biopsy-induced chordal rupture are also encountered in clinical practice. Secondary TR is far more common than primary TR. Secondary TR is marked by either right atrial or right ventricular enlargement leading to malcoaptation of the leaflets. Right atrial enlargement is associated with older age, atrial fibrillation, or remodeling in the setting of heart failure with preserved ejection fraction.^7^^,^^8^ Right ventricular (RV) dilatation and dysfunction are a consequence of increased afterload, which can be a consequence of the many etiologies of pulmonary hypertension including left heart disease, lung disease, and chronic thromboembolism. Ischemia and infarction can also lead to RV dysfunction.^9^ Patients with mild, moderate, and even severe TR are often asymptomatic. However, TR can also be harmful. Severe TR may lead to peripheral edema, hepatic congestion with cirrhosis, ascites, impaired renal function, and gut edema with intestinal malabsorption and cachexia. Fatigue, dyspnea, and exercise intolerance related to diminished cardiac output are common.^10^ Severe TR also leads to RV dilatation and dysfunction, which in turn exacerbates TR in a negative causal cycle. Advanced TR is associated with disabling heart failure, hospitalization, and death.^11^^,^^12^

Tricuspid repair is frequently performed in addition to mitral valve surgery, with excellent results and low mortality.^13^ However, given the high rate of recurrent infection following tricuspid valve replacement in the setting of endocarditis and intravenous drug use, early tricuspid literature endorsed valvulectomy without replacement, reserving tricuspid valve replacement for patients with refractory heart failure.^14^ Perhaps influenced by a pervasive attitude that TR is well-tolerated, isolated tricuspid valve surgery has been reserved for patients with refractory right heart failure, is rarely performed, and is associated with high morbidity and mortality.^15^^,^^16^ Patients undergoing isolated tricuspid valve surgery experience more in-hospital complications such as stroke and vascular complications, than their counterparts undergoing tricuspid valve surgery with concomitant cardiac surgery.^17^ Furthermore, isolated tricuspid valve surgery is associated with high healthcare resource utilization, in which index hospitalizations result in long lengths of stay, high hospital costs, and discharge to skilled nursing facilities.^17^^,^^18^ Medical therapy with diuretics has therefore been the mainstay of treatment for TR.^19^

## Tricuspid Valve Research Framework

The Tricuspid Valve Academic Research Consortium has developed standardized definitions of disease severity and trial endpoints that aim to enable meaningful comparisons between clinical trials.^20^ A 5-grade scale has been developed to categorize TR severity that stratifies “severe” TR into severe (grade 3), massive (grade 4), and torrential (grade 5).^21^ The reproducibility of this grading scale has not been validated, limiting confidence in comparisons of TR severity between trials.

Over the past few decades, patient values and preferences have been increasingly prioritized in healthcare research. Since 2019, the United States FDA has specifically endorsed patient-reported health status as an appropriate target endpoint for heart failure therapies.^22^ The Kansas City Cardiomyopathy Questionnaire (KCCQ) is a validated patient-reported outcome measure first developed in 2000 and is one of two heart failure specific patient-reported outcome questionnaires accepted by the U.S. FDA.^23^ The KCCQ is composed of 23 questions that address physical limitations, symptoms, self-efficacy, quality of life, and social limitations. The scores are totaled to provide a functional status score ranging from 0 to 100. Composite scores represent health status ranging from 0 to 24: very poor to poor; 25 to 49: poor to fair; 50 to 74: fair to good; and 75 to 100: good to excellent. By repeatedly asking the same questions over time, KCCQ captures the impact of heart failure on patients' lives and is strongly associated with clinical events over time. A change of 5 points in the KCCQ overall summary score is clinically meaningful; a change of 10 points represents a moderate to large improvement; and a change of 20 points is a large to very large improvement.^24^

The transcatheter tricuspid valve intervention trials have enrolled patients with different degrees of TR and at different stages of TR-induced right heart failure. The enrolled population of each trial is influenced by elements of the trial design, such as variable needs for diuretic stabilization and variability in anatomical selection. Some patients with large coaptation gaps and leaflets pinned by pacemaker leads traversing the tricuspid valve may be more amenable to tricuspid valve replacement than repair. Furthermore, both the TRISCEND II trial and the TRILUMINATE trial included nonrandomized registries that captured patients with more advanced disease. Beyond core lab-adjudicated TR severity, coaptation gaps and the presence of pacemakers are markers of TR severity and complexity. KCCQ scores, 6-minute walk distance (6MWD), and New York Heart Association (NYHA) class help define the severity of baseline TR-induced right heart failure.

## EVOQUE Tricuspid Valve Replacement and the TRISCEND Trials

The EVOQUE system has been previously described.^25^ Briefly, the EVOQUE valve consists of a nitinol frame, bovine pericardial leaflets, and a fabric skirt to minimize paravalvular regurgitation. It has 9 anchors that capture the native leaflets and expand into the subvalvular right ventricle for stabilization. The EVOQUE valve is delivered transfemorally through a 28 F delivery system. Transesophageal echo guidance with multiplane reconstruction, sometimes supplemented with 3D intracardiac echocardiography, is used to ensure leaflet capture of each anchor.

The TRISCEND feasibility study is a prospective, single-arm, multicenter study that evaluated the safety and performance of the EVOQUE system (NCT04221490). The 1-year results for 176 patients were promising.^26^ Two patients experienced valve embolization with surgical explantation, and 30-day cardiovascular mortality was 1.7%.^27^ As anticipated with tricuspid valve replacement, TR was reduced to ≤ mild in 98% of patients at 1 year, leading to increased stroke volume (by 11 ml) and increased cardiac output (by 0.6 l/min). At 1 year, NYHA class I or II was achieved in 93% of patients, the KCCQ score increased by 26 points, 6MWD increased by 56 meters, and right ventricular end diastolic diameter (RVEDD) decreased from 41 to 35 mm.

**Figure 3:**
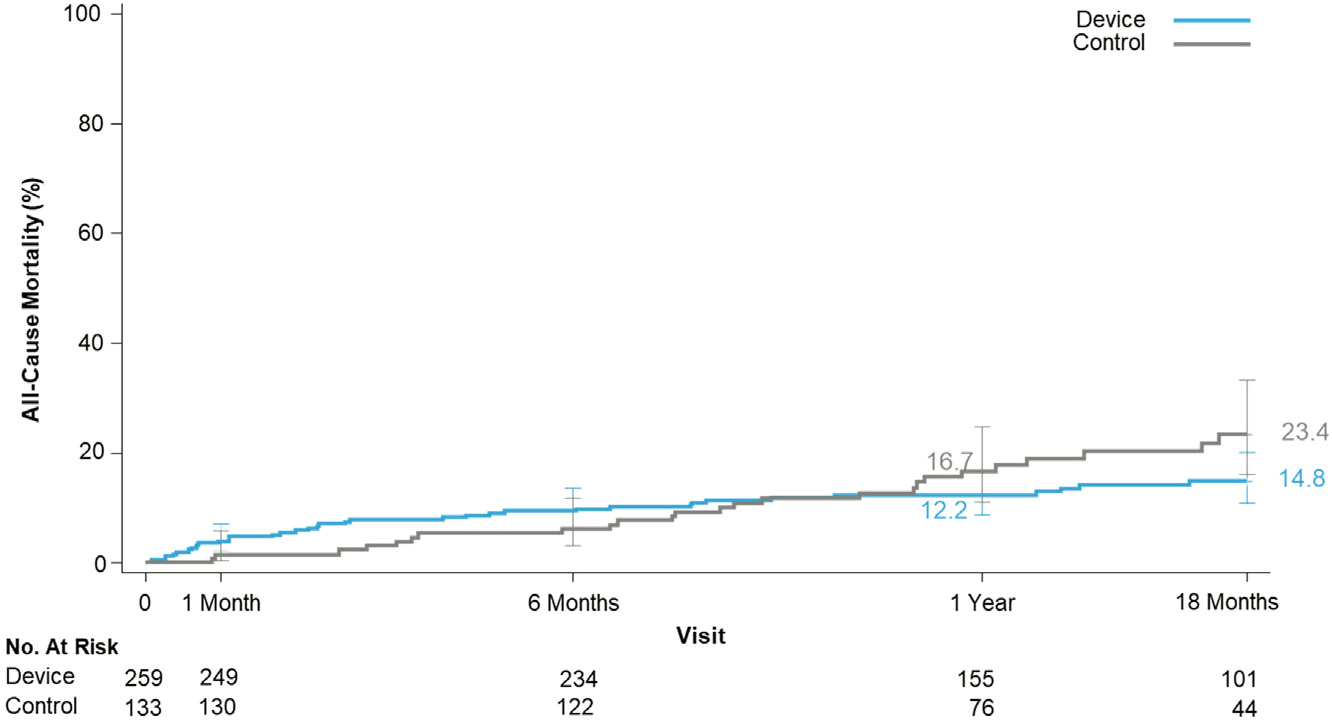
TRISCEND II all-cause mortality. This figure demonstrates the 1-year mortality results of the modified intention to treat population in TRISCEND II (all patients who had a study device attempted in the device group or who were randomized to the control group). Procedural hazard crosses at approximately 9 months, yielding a survival advantage with patients receiving the EVOQUE valve by 18 months. https://www.accessdata.fda.gov/cdrh_docs/pdf23/P230013B.pdf. Abbreviation: mITT, modified intention to treat.

The TRISCEND II pivotal trial is a prospective, multicenter trial that randomized patients with symptomatic severe TR to receive either EVOQUE transcatheter tricuspid valve replacement along with optimal medical therapy or optimal medical therapy alone in a 2:1 ratio. Distinguished by a novel 2-phase design based on the FDA Breakthrough Device Designation, the trial assesses 30-day safety and 6-month effectiveness endpoints for the first 150 patients, followed by a hierarchical composite endpoint of 1-year safety and effectiveness for the full 400-patient cohort in the second phase (Figure 2). TRISCEND II enrolled a high-risk and highly symptomatic population with complex anatomy (Table 1). Among patients randomized to devices, 79% had class III/IV heart failure, and the mean KCCQ score was 49. Pacemaker leads traversing the tricuspid valve were present in 37% of patients receiving an EVOQUE valve. As presented at TCT 2023, TRISCEND II met its prespecified safety performance goal. Major adverse events were experienced in 27% of patients including a 30-day cardiovascular mortality of 3.2%, a 11% risk of major bleeding, and a 15% risk of pacemaker. The KCCQ score increased by 22 points at 6 months with device compared to 4 points with medical therapy. Patients randomized to device improved their 6MWD by 11 meters compared to a 20 meter decline in patients randomized to medical therapy. The EVOQUE IFU includes Kaplan-Meier analysis of mortality with 145 patients reaching 18 months. While error bars are broad, the curves suggest an early procedural hazard that crosses at approximately 9 months to yield a small survival advantage with patients receiving the EVOQUE valve by 18 months (Figure 3).^5^ These preliminary results should be interpreted with caution pending longer-term follow-up of the entire TRISCEND II cohort.

**Table 1:** Baseline demographics, echocardiography, and quality of life metrics

	Feasibility trials			TRILUMINATE pivotal			International registries		TRISCEND II	
Study	TRISCEND	TRILUMINATE	CLASP TR	Device	Control	Single-arm registry	bRIGHT	PASTE	Breakthrough cohort device control	
Device	EVOQUE	TriClip	PASCAL	TriClip	Med Rx	TriClip	TriClip	PASCAL	EVOQUE	Med Rx
Sample size	176	85	65	175	175	100	511	235	96	54
NYHA III/IV (%)	75	75	71	59	55	59	80	90	79	70
RV pacemaker/ICD (%)	32		14	16	14	35	23	31	37	43
Coaptation gap (mm)				5.5		7.4		7		
KCCQ	47	52	53	56	54	55	45		49	50
6 MWD, meters	129	272	208	241	254			245	232	244

Abbreviations: 6 MWD, 6-minute walk distance; ICD, implantable cardioverter-defibrillator; KCCQ, Kansas City Cardiomyopathy Questionnaire; NYHA, New York Heart Association; RV, right ventricular.

## Transcatheter Tricuspid Valve Repair

### Feasibility Trials

The multicenter, prospective, single-arm TRILUMINATE and CLASP TR feasibility trials provided initial evidence of the safety and efficacy of transcatheter tricuspid edge-to-edge repair (T-TEER) and were used to model the pivotal trials, which share their same names. The feasibility studies demonstrated safe and efficacious procedures resulting in early, significant, and sustained improvements in KCCQ scores and 6MWD, diminished hospitalization, low mortality, and favorable right heart remodeling (Table 2).

**Table 2:** Echocardiogram and quality of life outcomes

1 y endpoints	Feasibility trials			TRILUMINATE pivotal			International registries		TRISCEND II‖	
Study	TRISCEND	TRILUMINATE	CLASP TR	Device	Control	Single-arm registry	bRIGHT†	PASTE†	Breakthrough cohort device control	
Device	EVOQUE	TriClip	PASCAL	TriClip	Med Rx	TriClip	TriClip	PASCAL	EVOQUE	Med Rx
Sample size (N)	176	85	65	175	175	100	511	235	Variable analysis	
Residual TR (%)	100	70	86	87	6	81	77	81	99‡	22‡
≤ Moderate	98	37	45	50	1	51	51	39	94‡	5‡
≤ Mild										
Mortality (%)	9	7	11	9	8	15		12∗	15§	23§
KCCQ change (mean)	+26	+20	+18	+12	+0.6	+15	+19		+22‡	4‡
6 MWD change (M)	+56	+31	+94	−8	−25			+54	+11‡	−20‡
RVEDD diameter (mm)	−6	−4	−3	−2	−1		−4		−6‡	−1‡

Abbreviations: 6 MWD, 6-minute walk distance; KCCQ, Kansas City Cardiomyopathy Questionnaire; RVEDD, right ventricular end diastolic diameter; TR, tricuspid regurgitation.

∗ 2-y endpoint.

† 30-d endpoint.

‡ 6-mo endpoint.

§ 18-mo Kaplan-Meier analysis.

‖ https://www.accessdata.fda.gov/cdrh_docs/pdf23/P230013B.pdf.

The TRILUMINATE feasibility trial included 85 patients treated with the TriClip device.^28^ TR was reduced to moderate or less in 70%, while a reduction of at least 1 grade was achieved in 87% of patients at 1 year. Single leaflet attachment occurred in 5 patients (7%). The KCCQ score increased by 20 points, and 6MWD increased by an average of 31 meters between baseline and 1 year. Clinical improvements occurred primarily within the first month of the index procedure, with no significant differences in NYHA functional class or 6MWD between 30-days and 1-year. Hospitalization decreased from 1.30 events per patient-year during the year prior to device implantation to 0.66 events per patient-year in the 2 years following the TriClip procedure.^29^ RVEDD decreased by 4 mm.

The PASCAL system was initially evaluated in the 65-patient CLASP TR feasibility trial. TR was reduced to moderate or less in 86% of patients at 1 year, and all patients benefitted from at least a 1 grade reduction in TR at 1 year.^30^^,^^31^ Rare complications included single leaflet attachment in 3 patients (4.6%). NYHA functional class improved with 92% in class I or II at 1 year; 6-minute walk distance increased by 94 m; and mean KCCQ scores improved by 18 points. Heart failure hospitalization was 56.4% lower in the year following T-TEER compared to the year preceding enrollment. Right atrial volume and RVEDD each decreased by approximately 12% at 1 year.

### TRILUMINATE Pivotal

The TRILUMINATE pivotal trial is the first randomized trial to evaluate and demonstrate the safety and efficacy of T-TEER.^1^ A total of 572 patients with symptomatic severe TR were randomly assigned in a 1:1 ratio to receive either T-TEER with the TriClip device or medical therapy. The primary endpoint was a composite of all-cause death, heart failure hospitalization, and improved quality of life, defined as a KCCQ increase of at least 15 points at 1 year. Results between the two groups were compared using a win ratio. Results were largely consistent between the initial 350 patient publication, the 572 patient TCT presentation, and the FDA panel briefing documents. TriClip T-TEER was safe and effective, with 98.3% of TriClip patients free from major adverse events at 30 days and 87% of TriClip patients achieving moderate or less core lab-adjudicated TR at 30 days. TRILUMINATE met its primary superiority endpoint with a win ratio of 1.48% favoring the T-TEER group. There were no differences between groups in the incidence of death or heart failure hospitalization. However, 50% of patients randomized to T-TEER vs. 26% of patients randomized to medical therapy achieved a 15-point increase in KCCQ-defined quality of life (QOL) driving the success of the primary composite endpoint.

**Figure 4:**
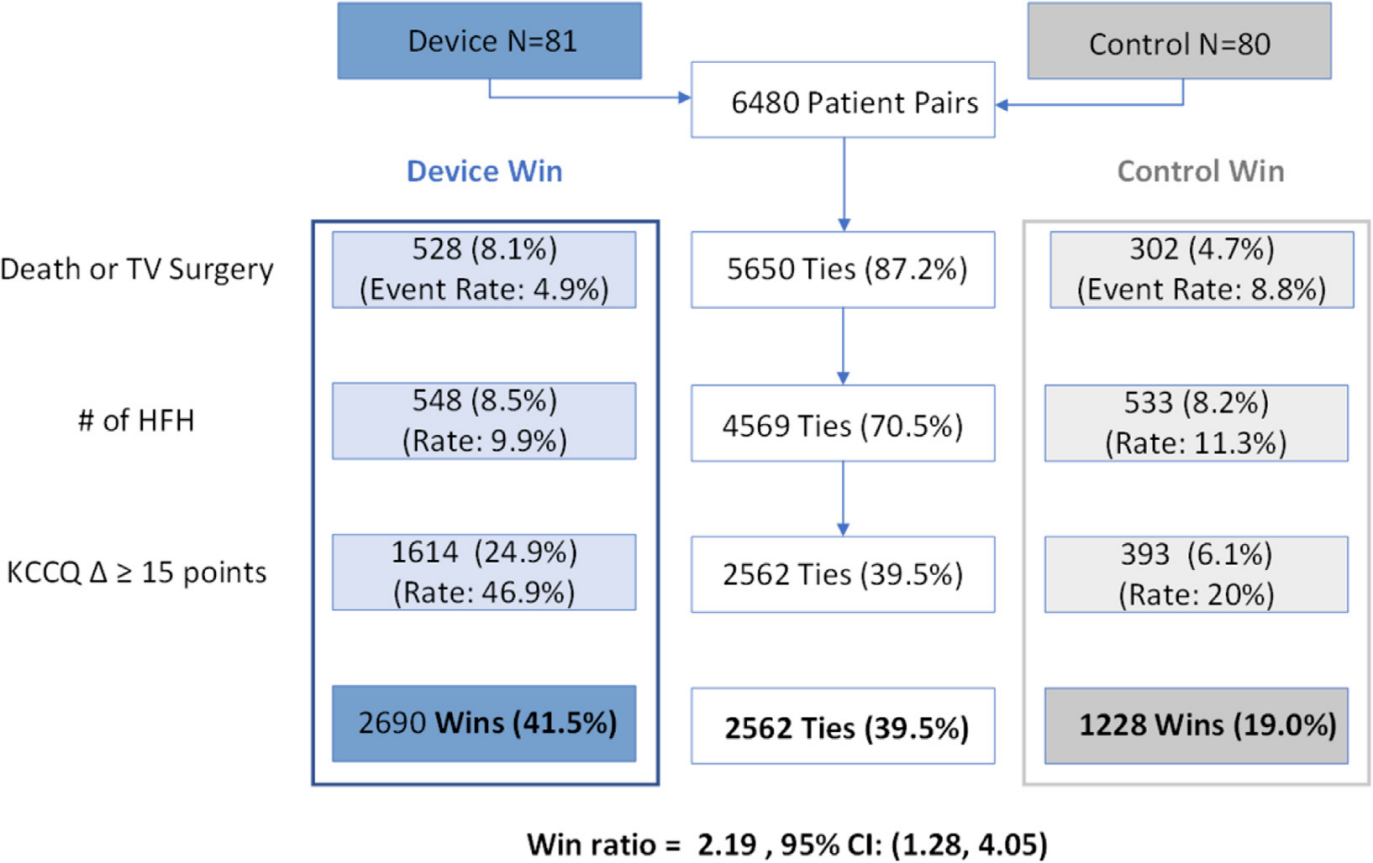
TRILUMINATE: Win ratio analysis of primary endpoint for sites that enrolled >10 subjects. This figure illustrates the favorable win ratio in the TEER group as compared with medical therapy. The primary endpoint was a hierarchical composite that included death from any cause or tricuspid-valve surgery; hospitalization for heart failure; and an improvement in quality of life as measured with the KCCQ. The win ratio analysis favored the TEER group in both the composite primary endpoint and each of its individual components. FDA Comment: The win ratio result of the primary endpoint for the group of sites that enrolled > 10 patients was more than two-fold higher vs. the group of sites that enrolled <10 patients. https://www.fda.gov/media/176088/download. Abbreviations: HFH, heart failure hospitalizations; KCCQ, Kansas City Cardiomyopathy Questionnaire; TEER, transcatheter edge-to-edge repair; TV, tricuspid valve.

TRILUMINATE represents the initial or very early T-TEER investigator experience and was conducted with minimal availability of 4D intracardiac echo. Patients randomized at high enrolling sites (≥10 patients) benefitted with a primary endpoint win ratio of 2.2%, more than two-fold higher than the group of sites that enrolled <10 patients (1.1%).^3^ Among patients enrolled at these high enrolling centers, death or tricuspid valve surgery was 4.9% with device and 8.8% among patients randomized to medical therapy (Figure 4).

**Figure 5:**
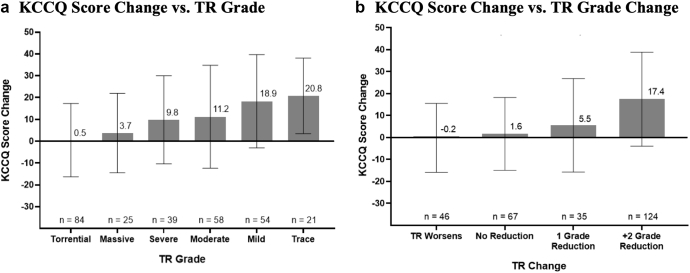
Association between KCCQ score and TR at 12 months. This figure illustrates the association between TR reduction and improvement in quality of life, as demonstrated in TRILUMINATE. Patients saw the greatest improvement in quality of life when TR was reduced by +2 grades (https://www.fda.gov/media/176088/download). Abbreviations: KCCQ, Kansas City Cardiomyopathy Questionnaire; TR, tricuspid regurgitation.

As a subjective endpoint, potentially influenced by the placebo effect, the clinical significance and validity of KCCQ have been questioned. As with most cardiology device trials, TRILUMINATE patients were not blinded to randomization or to the outcomes achieved by T-TEER. Study coordinators administering KCCQ surveys were blinded to randomization and procedure outcomes. The KCCQ quality-of-life score increased by a mean of 12.3 points in the T-TEER group, compared with 0.6 points in the control group (*p* < 0.001). Among patients with a baseline KCCQ score ≤50, 79% of patients in the T-TEER group benefitted from a 15-point or greater increase in KCCQ compared to 43.1% in the medically controlled group. The TRILUMINATE trial demonstrated a meaningful association between TR reduction and quality of life improvement. The mean KCCQ score increased by 19 points among patients with trace or mild residual TR but was essentially flat when TR grade remained unchanged (Figure 5).

An in-depth TRILUMINATE QOL analysis was presented at the October 2023 TCT by Suzanne Arnold with simultaneous publication.^32^ T-TEER patients were more likely to be alive and well with a KCCQ >60 at 1 year (75 vs. 46%, *p* < 0.001). A 10-point increase in KCCQ 1 month after T-TEER was associated with a 24% lower hazard for death (95% CI 0.64-0.90; *p* = 0.001), a 25% lower hazard for heart failure hospitalization (95% CI 0.64-0.89; *p* = 0.001), and a 26% lower hazard for the composite of death or heart failure hospitalization (95% CI 0.65-0.84; *p* < 0.001). The impact of T-TEER on 1-year health status was consistent across prespecified subgroups. The magnitude of the TRILUMINATE KCCQ-derived benefit, the sustained improvement from 30 days to 1 year, the association between the KCCQ improvement and reduced death and heart failure hospitalization, and the dramatic benefit among experienced operators suggest that T-TEER QOL benefit is not only a clinical effect rather than placebo but that the magnitude of benefit is also highly clinically meaningful.

TRILUMINATE included a prespecified imaging substudy in which a subgroup of patients was evaluated with cardiac computed tomography and magnetic resonance imaging at baseline and 30 days and cardiac computed tomography at 1 year. Preliminary results for 68 randomized patients were presented at the October 2023 TCT.^33^ Magnetic resonance imaging analysis confirmed the echo-derived TR reduction. The mean regurgitant volume decreased from 49 ml at baseline to 15 ml at 30 days following T-TEER, while regurgitant volume increased from 48 to 51 ml in patients randomized to medical therapy. Right ventricle volume decreased from 249 ml at baseline to 220 ml at 30 days and 214 ml at 1 year in patients randomized to T-TEER (*p* < 0.002) but was not significantly changed in control patients. RV volume changes are highly correlated to TR reduction, with an R value of 0.9.

**Figure 6:**
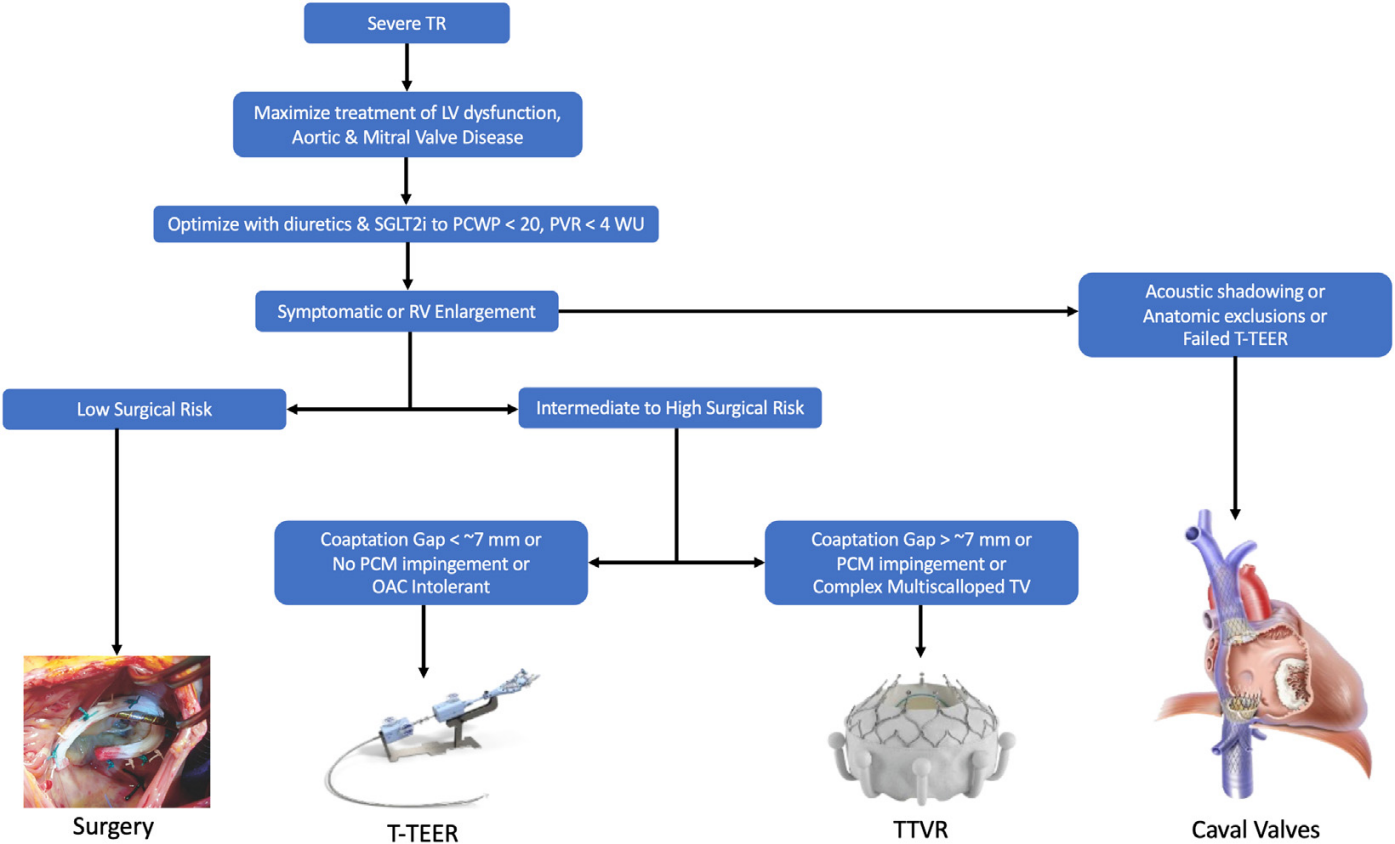
TR treatment algorithm. This figure illustrates a proposed algorithm for treating patients with severe TR. If present, LV dysfunction and valvular disease should be treated. Patients should then be medically optimized with diuretics and SGLT2i inhibitors, targeting a wedge pressure of less than 20 mmHg. If patients have severe pulmonary hypertension, they should be excluded from the treatment algorithm. If patients continue to have symptoms or signs of RV failure, surgical candidacy should be considered. For intermediate- or high-risk surgical patients, transcatheter therapies are appropriate. These patients are then subdivided by coaptation gap. Patients with a coaptation gap < 7 mm are better suited for TEER. TTVR is appropriate for intermediate- or high-risk surgical patients with large coaptation gaps. Caval valve implantation should be reserved for patients with acoustic shadowing, anatomic exclusions, or failed T-TEER. Abbreviations: LV, left ventricle; OAC, oral anticoagulant; PCM, permanent pacemaker; PCWP, pulmonary capillary wedge pressure; PVR, pulmonary vascular resistance; RV, right ventricle; SGLT2i, sodium/glucose cotransporter-2 inhibitor; TEER, transcatheter edge-to-edge repair; TR, tricuspid regurgitation; T-TEER, transcatheter tricuspid edge-to-edge repair; TTVR, transcatheter tricuspid valve replacement; TV, tricuspid valve; WU, Woods Units.

Given the success of the TR feasibility trials and the success of the COAPT trial, the failure of TRILUMINATE to demonstrate a reduction in death or heart failure hospitalization may seem disappointing on the surface.^34^ However, when comparing the magnitude of death and hospitalization between TRILUMINATE, TRISCEND II, and COAPT, it is apparent that TRILUMINATE was underpowered for these endpoints (Table 3). TRILUMINATE randomized patients who were less impaired according to NYHA class, 6MWD, and KCCQ score than the feasibility trials and the postapproval international registries (Table 1). This contributed to fewer deaths and heart failure hospitalizations than predicted by the trial’s statistical model. With the objective of diminishing postrandomization medical optimization, TRILUMINATE inclusion criteria included optimization and stabilization of medical therapy including oral diuretics. The most debilitated TR patients with advanced right heart failure often require IV diuretics and frequent paracentesis and could not be stabilized for inclusion in TRILUMINATE. TRILUMINATE candidates were reviewed by heart failure and case selection committees and were excluded with a pulmonary capillary wedge pressure >20 mmHg, extreme coaptation gaps (>10 mm), and difficult anatomy such as severe pacemaker-induced leaflet tethering. Patients with the largest coaptation gaps, the most severe TR, and the most difficult-to-manage right heart failure were ineligible for enrollment in the randomized cohort but were sometimes enrolled in the TRILUMINATE single-arm registry. An algorithm for managing patients with severe TR is proposed in Figure 6. Patients with diminished left ventricular systolic function should be optimized with guideline-directed medical therapy. Significant left-side valve disease should be treated. Patients should be optimally diuresed and excluded with severe pulmonary hypertension. Patients with persistent severe TR and symptoms or right ventricular enlargement should be considered for surgery or transcatheter intervention. Smaller coaptation gaps without significant pacemaker impingement may be well served with transcatheter tricuspid edge-to-edge repair, while larger coaptation gaps and leaflets pinned by right ventricular leads, particularly in patients tolerating oral anticoagulation may be best served with transcatheter tricuspid valve replacement.

**Table 3:** Endpoint frequency x intervention success = power

Trial endpoints	TRILUMINATE	TRISCEND II	COAPT
	1 y (N = 350)	Variable	2 y (N = 650)
Mortality	8%	23%∗	46%
HF related	3%		26%
Non-HF CV death	2%		17%
Non-CV death	3%		13%
Heart failure hospitalization	11%	38%‡	57%
Residual MR/TR			
Trace/mild	51%	94%†	77%
Moderate	38%	5%†	22%
>Moderate	11%	1%†	1%

Abbreviations: COAPT, Cardiovascular Outcomes Assessment of the MitraClip Percutaneous Therapy for Heart Failure Patients with Functional Mitral Regurgitation trial; CV, cardiovascular; HF, heart failure; MR, mitral regurgitation; TR, tricuspid regurgitation.

∗ 18-mo Kaplan-Meier analysis.

† 6 mo.

‡ Annualized HF Hospitalization (event/patient-year) https://www.accessdata.fda.gov/cdrh_docs/pdf23/P230013B.pdf.

### TRILUMINATE Single-Arm Registry

Among other factors, the case selection committee considered the ability to visualize all leaflets with transesophageal echocardiogram, coaptation gaps, and the interaction of pacemaker leads as they considered if patients were appropriate for inclusion in the randomized cohort. More challenging anatomies were selected for a nonrandomized registry rather than the randomized cohort. The nonrandomized registry results were presented at TCT in 2023.^2^ Patients enrolled in the single-arm registry were more likely to have pacemaker leads (36 vs. 16%), had larger coaptation gaps (mean 7.4 vs. 5.3 mm), had larger right ventricles (mean end diastolic dimension 4.0 vs. 3.7 cm), and were more likely to have grade 5 TR (74 vs. 50%). Despite challenging anatomy, moderate or less TR was achieved in 81% of registry patients, modestly lower than the 89% of patients treated with T-TEER in the randomized cohort. All-cause mortality (15 vs. 8.6%) and heart failure hospitalization (24 vs. 14.6%) were higher in the single-arm registry. Whether inclusion of these higher-risk and higher-degree TR patients in the randomized cohort may have demonstrated reduced mortality and/or heart failure hospitalization is open to speculation.

### Observational Registries

The TriClip and PASCAL devices have CE Mark approval in Europe, where they are available for commercial use. The commercial registries are not subjected to the rigid inclusion and exclusion criteria of the clinical trials and provide insights into contemporary T-TEER patient populations and the benefits derived from these populations. The commercial registries have included patients with more TR and more TR-induced morbidity than TRILUMINATE, and these patients have benefitted from a greater improvement in KCCQ scores than TRILUMINATE (Tables 1 and 2). Like the TRILUMINATE single-arm registry, the potential of T-TEER to reduce mortality and heart failure hospitalization is more plausible in the more impaired registry populations but remains unproven. The bRIGHT registry is an ongoing, prospective registry with 26 European centers and core-lab adjudication of 511 patients at the latest presentation.^35^ The mean baseline KCCQ score is 44.5 in bRIGHT, compared to 56 in TRILUMINATE, and 80% have class III/IV heart failure compared to 59% in TRILUMINATE (Table 1). The PASTE registry is an ongoing retrospective, observational study examining patients undergoing T-TEER with the PASCAL system in Europe, with a target enrollment of 1000 patients.^36^ One-year data in 380 patients were presented in May 2023 at EuroPCR. At baseline, 90% of patients described class III or IV NYHA symptoms.

### CLASP II TR

The CLASP II TR Trial is currently randomizing patients in a 2:1 ratio between the PASCAL system and medical therapy (NCT04097145). With a larger population, CLASP II TR may have increased power to show efficacy when compared with TRILUMINATE. The primary endpoint is a composite of all-cause mortality, right ventricular assist device implantation, heart transplantation, tricuspid valve intervention, heart failure hospitalizations, and KCCQ-defined quality of life improvement after 24 months. The CLASP II TR Trial includes a single-arm registry cohort of patients who are not eligible for randomization. Randomization vs. medical therapy is increasingly difficult with FDA-approved devices. The regulatory pathway for the PASCAL system in the treatment of TR is currently unclear.

## Conclusion

The EVOQUE system was the first transcatheter tricuspid device to receive FDA approval, followed swiftly by the TriClip. The EVOQUE valve addresses complex tricuspid anatomy including large coaptation gaps and pacemaker leads with 98% of patients having mild or less TR. TRISCEND II study results have demonstrated that the EVOQUE tricuspid valve replacement system in patients with advanced heart failure results in marked improvement in QOL and favorable trends in the primary composite endpoints, including all-cause mortality, heart failure hospitalization, and 6MWD. Presentation of the full cohort of TRISCEND II pivotal trial patients is forthcoming. The TRILUMINATE Pivotal trial has demonstrated strong evidence supporting T-TEER over medical therapy to reduce TR and improve quality of life with favorable RV remodeling. Just as the most impaired secondary mitral regurgitation patients derive the greatest benefit from COAPT, the most impaired TR patients derive the greatest magnitude of benefit from T-TEER.^37^

The tricuspid valve has long been considered the “forgotten valve.” TR can be well tolerated for many years but may also progress with disabling right heart failure and death. Transcatheter tricuspid valve replacement and repair have the potential to address the unmet needs of patients suffering with TR and right heart failure while restoring and preserving RV function with improved survival. Multidisciplinary heart teams including structural, imaging, and heart failure cardiologists and cardiac surgeons should help guide future research and the care of individual patients.

## Funding

The authors have no funding to report.

## Disclosure Statement

The authors report no conflict of interest.
